# Editorial: The Relationship Between Cancer Predisposition and Primary Immunodeficiency

**DOI:** 10.3389/fimmu.2019.01781

**Published:** 2019-07-30

**Authors:** Fabian Hauck, Andrew R. Gennery, Markus G. Seidel

**Affiliations:** ^1^Pediatric Immunology and Rheumatology, Department of Pediatrics, Dr. von Hauner Children's Hospital, University Hospital, Ludwig-Maximilians-University Munich, Munich, Germany; ^2^Department of Paediatric Immunology + HSCT, Great North Children's Hospital, Institute of Cellular Medicine, Newcastle University, Newcastle upon Tyne, United Kingdom; ^3^Research Unit for Pediatric Hematology and Immunology, Division of Pediatric Hemato-Oncology, Department of Pediatric and Adolescent Medicine, Medical University Graz, Graz, Austria

**Keywords:** inborn error of immunity, IEI, primary immunodeficiency, PID, cancer predisposition syndrome, CPS

## Introduction

The risk of malignancies is higher in patients with genetically determined inborn errors of immunity (IEI) than in the general population (1, 2). However, the degree of tumor predisposition and the underlying cellular and molecular mechanisms vary through the categories of IEI (3, 4). In addition to perturbed tumor immune surveillance in IEI and chronic inflammation or infections, the molecular defect *per se* that causes IEI may predispose to tumorigenesis (5). This suggests that malignancy in IEI may not merely be a consequence of immune deficiency, but occur in parallel to or even precede immune deficiency. Furthermore, in genetically determined DNA repair deficiencies the particular IEI may be perceived as an add-on to tumor predisposition (6–8). Additionally, deregulation of epigenetic factors related to IEI or its treatment such as alterations of the microbiome can contribute to tumor predisposition (9, 10).

The Research Topic covers many aspects of this increasingly appreciated clinical and basic scientific field. Furthermore, based on presentations and discussions directly or indirectly related to the process of the present collection, additional initiatives were launched and are ongoing. The 2019 focused meeting of the European Society of Immunodeficiencies in Brussels—“Malignancy and PID” (https://esidmeeting.org)—exemplifies this bringing together of specialists.

## Composition

### Large Cohort Studies

Two nationwide studies of common variable immunodeficiency (CVID) and one large international study of patients with haploinsufficiency of CTLA4 were conducted and provide insight into the cancer risk in these relatively frequent and highly relevant entities (Egg et al.; Kralickova et al.; Pulvirenti et al.). The need for awareness and appropriate screening measures is highlighted.

### Systematic Review

A comprehensive meta-review on lymphoid malignancies IEI structured according to the classification of the international union of immunological societies (IUIS), gives a clear, detailed, and helpful overview of the current knowledge, types, and distribution of B and T cell lymphoid malignancies associated with IEI (Riaz et al.).

### Perspective

The “*Current understanding and research priorities*…” in the challenging field of malignancies in IEI were defined, discussed, prioritized, and summarized by an interdisciplinary working group consisting of hematologists, oncologists, immunologists, tumor biologists, and geneticists and are presented (Bomken et al.).

### Small Cohort Studies, Single Entity or Patient Reports, or Reviews

A diverse collection of relevant clinical observations was reported, ranging from a single center long-term experience of malignancies in IEI (Maffeis et al. *in press*), over lymphomagenesis in STK4 deficiency or ataxia telangiectasia, variable phenotypes of Cernunnos/XLF deficiency, as well as the study of clinical and biological signs of immune deficiency in patients with the cancer predisposition syndrome *constitutional mismatch repair deficiency* (Recio et al.; Schipp et al.; Tatfi et al.; Tesch et al.). Additionally, the risk of malignancies in patients with secondary immunodeficiency due to immunosuppressive drugs in the framework of solid organ transplantation is reported, aiming at identifying specific drug-dependent mechanisms and risk factors (Cangemi et al.).

### Conceptual Review and Mini Reviews

One large conceptual review embedded the “*Closely related concepts*” of IEI and cancer predisposition syndromes into an integrative framework (Haas), while smaller (mini) reviews focused on tumor profiles in IEI, in Down Syndrome, or on common genetic bases of cancer and IEI (Derpoorter et al.; Satge; Satge and Seidel).

### Basic and Methodological Research

A mouse study on the effects of the loss of JAK1 on innate immunity, with a potential consequence of reduced tumor surveillance (Witalisz-Siepracka et al.), and a methodological study on improved early detection of the transformation risk in severe congenital neutropenia (Klimiankou et al.) complete the spectrum of articles.

## Conclusions and Perspectives

While it is evident that the concept of cancer predisposition and immune deficiency as opposite sites of the same genetic coin is still in its infancy, we at this point in time can state that it is born and rapidly growing.

We envision that in the era of systems biology and “omics” technologies there will be major advances not only in basic science, but as well in the ways geneticists, tumor biologists, immunologists, and oncologists will work together to finally improve diagnosis, treatment and patient outcome both in the sense of overall survival and in terms of quality adjusted life years and reproductivity.

## Author Contributions

FH and MS drafted the article. AG approved it.

### Conflict of Interest Statement

The authors declare that the research was conducted in the absence of any commercial or financial relationships that could be construed as a potential conflict of interest.
